# Technical Note: Characterization of the new microSilicon diode detector

**DOI:** 10.1002/mp.13710

**Published:** 2019-07-31

**Authors:** Ann‐Britt Schönfeld, Daniela Poppinga, Rafael Kranzer, Rudy Leon De Wilde, Kay Willborn, Björn Poppe, Hui Khee Looe

**Affiliations:** ^1^ University Clinic for Medical Radiation Physics Medical Campus Pius Hospital, Carl von Ossietzky University Oldenburg Germany; ^2^ PTW‐Freiburg Freiburg Germany; ^3^ University Clinic for Gynecology, Pius Hospital Oldenburg Germany; ^4^ Clinic for Radiation Therapy Pius Hospital Oldenburg Germany

**Keywords:** output correction factor, silicon diode, small‐field, volume effect

## Abstract

**Purpose:**

Dosimetric properties of the new microSilicon diode detector (60023) have been studied with focus on application in small‐field dosimetry. The influences of the dimensions of the sensitive volume and the density of the epoxy layer surrounding the silicon chip of microSilicon have been quantified and compared to its predecessor (Diode E 60017) and the microDiamond (60019, all PTW‐Freiburg, Germany).

**Methods:**

Dose linearity has been studied in the range from 0.01 to 8.55 Gy and dose‐per‐pulse dependence from 0.13 to 0.86 mGy/pulse. The effective point of measurement (EPOM) was determined by comparing measured percentage depth dose curves with a reference curve (Roos chamber). Output ratios were measured for nominal field sizes from 0.5 × 0.5  cm^2^ to 4 × 4 cm^2^. The corresponding small‐field output correction factors, *k*, were derived with a plastic scintillation detector as reference. The lateral dose–response function, *K*(*x*), was determined using a slit beam geometry.

**Results:**

MicroSilicon shows linear dose response (*R* ^2^ = 1.000) in both low and high dose range up to 8.55 Gy with deviations of only up to 1% within the dose‐per‐pulse values investigated. The EPOM was found to lie (0.7 ± 0.2) mm below the front detector’s surface. The derived *k* for microSilicon (0.960 at *s*_eff_ = 0.55 cm) is similar to that of microDiamond (0.956), while Diode E requires larger corrections (0.929). This improved behavior of microSilicon in small‐fields is reflected in the slightly wider *K*(*x*) compared to Diode E. Furthermore, the amplitude of the negative values in *K*(*x*) at the borders of the sensitive volume has been reduced.

**Conclusions:**

Compared to its predecessor, microSilicon shows improved dosimetric behavior with higher sensitivity and smaller dose‐per‐pulse dependence. Profile measurements demonstrated that microSilicon causes less perturbation in off‐axis measurements. It is especially suitable for the applications in small‐field output factors and profile measurements.

## 1 Introduction

Silicon diode detectors are generally used for dosimetric characterization of radiation beams, where high spatial resolutions are required, such as small‐field output factor and profile measurements. The sensitive volumes of most commercial silicon diode detectors have a diameter of around 1 mm, so that their signals are minimally perturbed by the geometrical volume‐averaging effect.^1^, ^2^ However, the enhanced density detector’s components, such as the silicon chip and its surrounding layers, will cause these detectors to over‐respond in small fields along the central axis. This so‐called density effect is a result of the perturbation of secondary electrons’ fluence by the enhanced density components in situations where lateral secondary electrons equilibrium is not established.^3^, ^4^, ^5^, ^6^ Consequently, the resulting small field output correction factors for silicon diode detectors are less than unity.^7^

In contrast, the silicon diode detector is also known to over‐respond in situations with increased contribution from low‐energetic scattered photons due to the larger photo‐effect interaction cross sections of silicon with *Z* = 14, as compared to water (*Z*_eff_ = 7) and air (*Z*_eff_ = 7.54). Previous studies have also shown that silicon diode detectors exhibit non‐negligible dose‐rate or dose‐per‐pulse dependence.^8^

In this work, the dosimetric properties of a new unshielded silicon diode detector, microSilicon (60023), are studied. Compared to its predecessor Diode E (60017), the new microSilicon has been optimized to achieve higher dose stability, lower dose‐per‐pulse dependence, smaller sensitivity to temperature variation, and smaller detector‐to‐detector variation. Furthermore, the density of the epoxy layer surrounding the silicon chip has been reduced. The dosimetric behavior of the microSilicon has been compared to that of its predecessor and to the microDiamond (60019).

## 2 Materials and methods

The physical properties of the new microSilicon with a slightly larger diameter (1.5 mm) and a more water‐equivalent epoxy layer surrounding the silicon chip are compared to the predecessor Diode E and the microDiamond (Table 1). The schematic drawing of the microSilicon is shown in Fig. S1 in the supplementary material.

**Table 1:** Comparison of detector data between the microSilicon (60023), the Diode E (60017), and the microDiamond (60019) according to the manufacturer’s detector data sheets.

	Diameter of sensitive volume/ mm	Depth of sensitive volume/μm	Sensitive volume/ mm^3^	Density of epoxy/ g/cm^3^
60023	1.5	18	0.032	1.15
60017	1.2	28	0.032	1.77
60019	2.2	2	0.008	1.09

In this study, two microSilicon detectors (60023 serial numbers: 151810 and 151811) have been investigated. Five other detectors have been used either for comparison purposes or to serve as reference detectors: silicon Diode E (60017), microDiamond (60019), 0.3 cm^3^ Semiflex ionization chamber (31013), a Roos plane‐parallel chamber (34001), all from PTW‐Freiburg, Germany, and a plastic scintillation detector W1 (Standard Imaging, Middleton WI, USA).

### 2.1 Dose linearity

Linearity measurements were performed at a Siemens Primus linear accelerator (Siemens Healthcare, Erlangen, Germany). Two microSilicon detectors and its predecessor Diode E were placed in a depth of 2.5 cm within a stack of 10‐cm thick solid water (RW3, PTW‐Freiburg, Germany) slabs. The source‐to‐detector distance (SDD) was 100 cm and a nominal field size of 10 × 10 cm^2^ was used. The detectors were irradiated using a 6‐MV photon beam with MU values from 1 to 1000 (0.01–8.55 Gy). To correct for possible variations in the accelerator output, an ionization chamber (Semiflex 31013) was used as the reference detector at the same point of measurement.

### 2.2 Dose‐per‐pulse dependence

Using the same setup as the linearity measurements, the SDD varied between 60 and 155 cm to achieve DPP values at the point of measurement from 0.13 mGy/pulse to 0.86 mGy/pulse using a 10‐MV photon beam with a pulse period of 6.4 ms. The Semiflex chamber was used as the reference detector to measure the absolute dose, for which the DPP‐dependent recombination loss has been corrected according to the method of Bruggmoser et al*.* 9 and DIN 6800‐2.^10^

### 2.3 Effective point of measurement

The effective point of measurement (EPOM) of the microSilicon has been determined using the methods described in Looe et al.^11^ by comparing the percentage depth dose (PDD) curve measured with the microSilicon and a reference curve obtained using a Roos plane‐parallel plate chamber. The EPOM of the Roos chamber is located at 1.5 ± 0.1 mm below the front surface.^11^ Measurements were performed in a water phantom (MP3‐M, PTW‐Freiburg, Germany) using a 6‐MV photon beam with two field sizes, 4 × 4 cm^2^ and 10 × 10 cm^2^. The source‐to‐surface distance (SSD) was 100 cm. The microSilicon was positioned with its outer front surface aligned to the water surface. The shift between the thereby obtained PDD and the reference PDD indicates the shift of the EPOM from the detector’s front surface.

### 2.4 Small‐field output correction factors

Output ratios (OR) were measured at a Siemens Artiste linear accelerator (Siemens Healthcare, Erlangen, Germany) with a 6‐MV photon beam at nominal field sizes from 0.5 × 0.5 cm^2^ to 4 × 4 cm^2^ using two microSilicon detectors, one Diode E, and one microDiamond. All detectors were positioned with their EPOM at the measurement depth of 10 cm in a water phantom (MP3‐M) with a SSD of 90 cm. A plastic scintillation detector W1 was used as the reference detector to obtain the field output factor (OF) and the corresponding small‐field output correction factor, *k*, according to TRS 483.^7^ Cerenkov corrections were performed according to the methods described in Burke et al.^12^ and Poppinga et al.^1^ Each detector was centered individually at each measured field size using the CenterCheck tool in the software MEPHYSTO mc^2^ (PTW‐Freiburg, Germany). The effective dosimetric field size length, *s*_eff_, was computed according to TRS 483.^7^

The volume‐averaging effect was investigated separately by first measuring the two‐dimensional dose profiles of the investigated field sizes using EBT3 films. The calibration and processing of the films were done as described in Poppinga et al.^13^ The measured signals of the detectors subjected to volume‐averaging effect, *M*_VAE_, were computed by averaging the dose values within the projection of the sensitive volume of the detectors on a plane orthogonal to the beam axis, which are all circular areas with diameters of 1.2 mm (Diode E 60017), 1.5 mm (microSilicon 60023), and 2.2 mm (microDiamond 60019). The dose values, *D*, were obtained by averaging the dose profiles over a circular area with 1 mm diameter. The volume averaging correction factors, *k*_vol_, were computed as the ratio *D*/*M*_VAE._

### 2.5 Lateral dose–response functions

The lateral dose–response function of a detector acts as the convolution kernel *K(x,y)* transforming the dose profile *D(x,y)* into the measured signal profile *M(x,y)* according to the convolution model^14^, ^15^, ^16^:

(1) $$M\left(x,y\right)=D\left(x,y\right)*K\left(x,y\right)$$

The area‐normalized function *K(x,y)* characterizes the detector’s volume effect, which comprises the geometrical volume averaging effect due to the finite extension of the detector’s dimensions and the perturbation of electron fluence due to the electron density of the detector’s components differing from that of normal water.

The one‐dimensional lateral dose–response function, *K(x)*, of the microSilicon was determined according to the methods described in Poppinga et al.^13^ utilizing a slit beam geometry. The detectors were scanned along the narrow side of the slit beam (0.05 cm wide) using a 6‐MV photon beam at a Siemens Primus linear accelerator to obtain *M(x)*. Additionally, the dose profile *D(x)* was measured using EBT3 films. The *K(x)* of the Diode E was determined similarly for comparison. The procedure for the deconvolution of eq. (1) to obtain *K(x)* from the measured *M(x)* and *D(x)* was performed according to Poppinga et al.^13^ described in the supplementary material.

### 2.6 Profile measurements

Profile measurements were performed using a microSilicon, a Diode E, and a microDiamond at a Varian TrueBeam linear accelerator (Varian Medical Systems, Palo Alto CA, USA). The 6‐MV beam profile of a 0.6 × 0.6 cm^2^ and a 1.6 × 1.6 cm^2^ field was scanned at a SSD of 95 cm in 5 cm water depth.

## 3 Results

### 3.1 Dose linearity

Both the Diode E and the new microSilicon detectors show linear dose response within the range of 0.01–8.55 Gy. The coefficient of determination *R* ^2^ was determined to be 1.000 for the three detectors (see Fig. S2 in the supplementary material). Furthermore, the dose response of the microSilicon is found to be about twice as high as the dose response of its predecessor.

### 3.2 Dose‐per‐pulse dependence

In the range of 0.13 mGy/pulse to 0.86 mGy/pulse, both the microSilicon and the microDiamond show negligible DPP dependence, whereas the response of the predecessor Diode E increases by up to 3% over the investigated DPP range. The relative DPP dependence of the microSilicon, as compared to the Diode E and the microdiamond, is shown in Fig. S3 in the supplementary material.

### 3.3 Effective point of measurement

The PDD curves obtained using the microSilicon detectors were shifted along the depth axis to achieve the best agreement with the reference curve obtained with the Roos chamber by minimizing the square of the relative difference between the PDD curves for the field size 10 × 10 cm^2^. The optimal shift was found to be 0.7 ± 0.2 mm, which indicates that the EPOM of the microSilicon lies 0.7 ± 0.2 mm below the outer front surface. The same shift was obtained using a field size of 4 × 4 cm^2^.

### 3.4 Small‐field output correction factors

The derived small‐field output correction factors, *k*, for the microSilicon detectors, Diode E. and microDiamond are presented in Table 2. All detectors exhibit correction factors less than unity for *s*_eff_ below 2 cm, showing an over‐response behavior. The correction factors of the two exemplars of the microSilicon show only a maximum deviation of 0.3 % from each other.

**Table 2:** Small‐field output correction factors, *k*, of the two microSilicon detectors (60023, serial numbers: 151810 and 151811), the Diode E (60017), and the microDiamond (60019) investigated in this work.

Small‐field output correction factor *k*				
Effective field size length *s*_eff_/ cm	60023 151810	60023 151811	60017	60019
0.55	0.960 ± 0.019	0.961 ± 0.019	0.929 ± 0.019	0.956 ± 0.019
0.63	0.969 ± 0.010	0.966 ± 0.010	0.941 ± 0.010	0.962 ± 0.010
0.81	0.989 ± 0.010	0.989 ± 0.010	0.973 ± 0.010	0.977 ± 0.010
1.10	0.997 ± 0.010	0.999 ± 0.010	0.988 ± 0.010	0.988 ± 0.010
1.59	0.999 ± 0.010	0.999 ± 0.010	0.996 ± 0.010	0.993 ± 0.010
2.00	0.998 ± 0.010	1.000 ± 0.010	0.999 ± 0.010	0.995 ± 0.010
3.00	1.003 ± 0.010	1.003 ± 0.010	1.003 ± 0.010	1.001 ± 0.010
4.00	1.000 ± 0.010	1.000 ± 0.010	1.000 ± 0.010	1.000 ± 0.010

**Figure 1:**
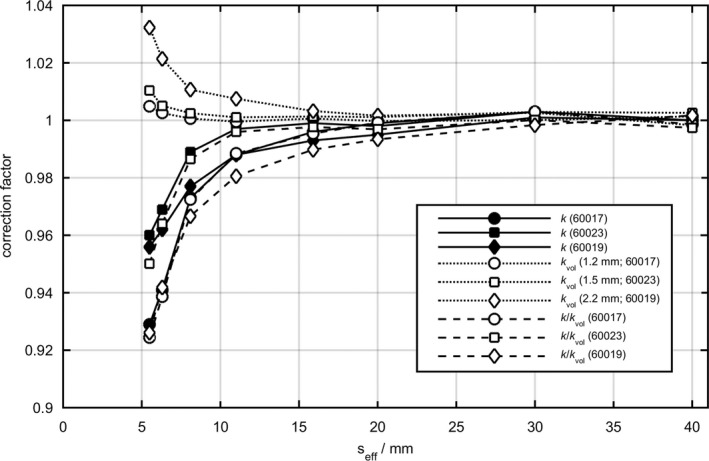
Comparisons of small‐field correction factors, *k*, volume‐averaging correction factors, *k*_vol_, and the ratios *k*/*k*_vol_ for the Diode E (60017), microSilicon (60023), and microDiamond (60019).

The correction factors for volume‐averaging, *k*_vol_, for the microSilicon, Diode E, and microDiamond are presented in Fig. 1. The remaining perturbation effects, except for the volume averaging effect, were derived from the ratios *k*/*k*_vol_.

### 3.5 Lateral dose–response functions

**Figure 2:**
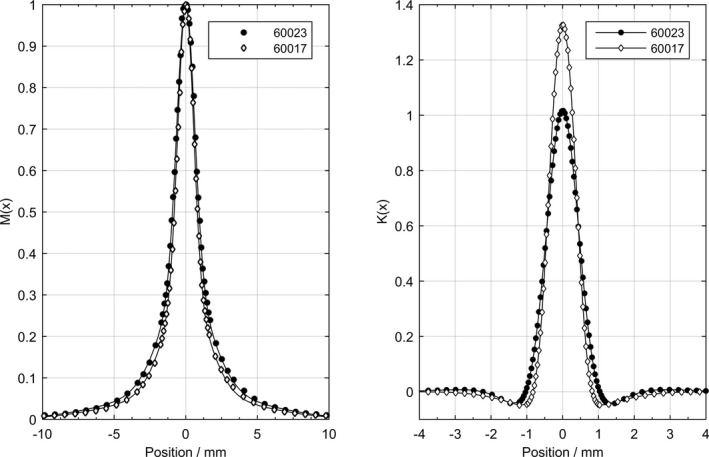
Left: Relative slit profiles *M(x)* measured with the microSilicon (60023) and the Diode E (60017) fitted by sums of three centered one‐dimensional Gaussian functions. Right: The area‐normalized one‐dimensional lateral dose–response functions, *K(x)*, of the microSilicon (60023) and the Diode E (60017).

The relative slit profiles *M(x)* measured with the microSilicon and the Diode E are fitted by sums of three centered one‐dimensional Gaussian functions (Fig. 2, left panel). The area‐normalized lateral dose–response functions, *K(x)*, of the microSilicon and the Diode E are shown in Fig. 2 (right panel). The full width at half maximum of the *K(x)* of the Diode E obtained in this study and the *K(x)* published previously by Poppinga et al*.* 17 agree within 0.1 mm. The fitting parameters $A_{i,K}$ and $\sigma_{i,K}$ according to eq. (S2) are listed in Table S1 in the supplementary material.

### 3.6 Profile measurements

The relative dose profiles measured with the microSilicon and the Diode E at a Varian TrueBeam linear accelerator for a field size of 0.6 × 0.6 cm^2^ and 1.6 × 1.6 cm^2^ are compared against microDiamond’s measurements, which has been shown to cause the least perturbation in off‐axis measurements^18^ (results are presented in Fig. S4 in the supplemental material). While the profiles measured using the Diode E show the steepest dose gradient with up to 4% deviation from the microDiamond’s measurements, the profiles measured using the new microSilicon exhibit maximum 1% deviation from the microDiamond’s measurements.

## 4 Discussion

The new microSilicon detector shows a linear dose response in the investigated range of 0.01–8.55 Gy. Its detector response is twice as high as the predecessor model Diode E even though their sensitive volumes are equal (see Table 1). While the response of the Diode E changes by up to 3% in the DPP range of 0.13 mGy/pulse to 0.86 mGy/pulse, the microSilicon exhibits a lower DPP dependence comparable to that of the microDiamond, where the detector’s response changes by less than 1%. The DPP dependence of the microDiamond detector agrees with the results published by Brualla‐González et al.^19^ For the axial orientation, the EPOM of the microSilicon was found to lie 0.7 ± 0.2 mm below the detector’s outer front surface, which is comparable to the manufacturer’s specification of 0.8 mm.

The small‐field output correction factors of the microSilicon, Diode E, and microDiamond are less than unity for *s*_eff_ smaller than 2 cm, that is, all these detectors over‐respond in small field sizes. At these field sizes, the microSilicon requires less correction than the predecessor Diode E and the correction factors are comparable to those obtained for the microDiamond detector. At the smallest field size investigated in this study (*s*_eff_ = 0.55 cm), the correction factor for the microSilicon amounts to 0.960 while the correction factor for the Diode E was found to be 0.929. For small fields, only detectors requiring less than 5% correction are recommended in the recently published TRS 483.^7^ In this study, it has been demonstrated that both the new microSilicon and the microDiamond fulfill this guideline down to the smallest investigated field size with *s*_eff_ = 0.55 cm, while the correction for the Diode E is larger than 5% in the two smallest field sizes investigated. The correction factors of Diode E and microDiamond obtained in this work agree to the TRS 483^7^ data within 1% and 0.5%, respectively.

As shown in previous studies,^4^, ^6^, ^20^ the factors *k*/*k*_vol_ of the silicon diode detectors are dominated by the density effect with values less than unity, while the microSilicon exhibits less density perturbation mainly owed to the reduced density of the epoxy layers. For the microDiamond detector, it has been recently shown that the observed over‐response is partly caused by radiation‐induced charge imbalance in the detector’s components.^21^ Nevertheless, this over‐response is largely compensated in small‐fields by the volume‐averaging effect, resulting in overall correction factors, *k,* similar to that of microSilicon.

Due to the larger diameter of its sensitive volume, the *K(x)* of the microSilicon is slightly wider with a lower maximum value than the *K(x*) of the Diode E. In contrast, negative values outside the border of the detector’s dimensions are observed for both detectors, which are caused by the enhanced density detector components, such as the silicon chip and the surrounding epoxy layer. Nevertheless, the amplitude of these negative values is lower for the microSilicon indicating an improved water‐equivalent of the new epoxy layer. For relative profile measurements, the microSilicon also shows less deviation than the Diode E from the microDiamond detector, which has been demonstrated by Francescon et al.^18^ to provide the least perturbation at dose gradient regions.

## 5 Conclusions

In this work, the dosimetric properties of the new microSilicon detector have been characterized with focus on its application in small‐field dosimetry. Compared to its predecessor, the microSilicon shows smaller DPP dependence and the correction factors for small‐field output factor measurements are closer to unity. The measured profiles using the microSilicon also show better agreement to the microDiamonds’s measurements in the penumbra regions. Overall, the microSilicon has been demonstrated to possess improved characteristics compared to the predecessor for use in small‐field dosimetry.

## Conflict of interest

Daniela Poppinga and Rafael Kranzer are employees of PTW‐Freiburg. The other authors have no conflict of interest to report.

## Supporting information


**Figure S1: **Schematic cross‐section of the microSilicon. All dimensions given in the unit mm. The silicon chip is shown in dark grey, the detector housing in light grey and the epoxy encapsulation in white.
**Figure S2: **Linearity of the dose response of the microSilicon (60023) and Diode E (60017). The left panel shows the whole range of dose applied (0.01 Gy to 8.55 Gy); the right panel shows the low dose range up to 0.1 Gy.
**Figure S3: **Relative DPP dependence of the microSilicon (60023), the Diode E (60017) and the microDiamond (60019) presented as the ratio of the detector’s signal and the Semiflex ionization chamber’s signal that has been corrected for recombination loss.
**Figure S4: **Comparison between the cross‐plane profiles measured using microSilicon, Diode E and microDiamond at a Varian TrueBeam linear accelerator using a field size of 0.6 × 0.6 cm² (left) and 1.6 × 1.6 cm² (right). The percentage deviations of the microSilicon and Diode E from the microDiamond’s profile are shown at the bottom.
**Table S1: **Fitting parameters for K(x) according to eq. (S2) for the microSilicon (60023) and the Diode E (60017). *σ_i_* in mm.
