# Supplementary material for: Technical Note: Characterization of the new microSilicon diode detector
Source: Med Phys. 2019 Jul 31;46(9):4257–62. doi: 10.1002/mp.13710 (PMC6852691; doi:10.1002/mp.13710)
Supplement: Supplementary file 1 — Figure S1: Schematic cross‐section of the microSilicon. All dimensions given in the unit mm. The silicon chip is shown in dark grey, the detector housing in light grey and the epoxy encapsulation in white. Figure S2: Linearity of the dose response of the microSilicon (60023) and Diode E (60017). The left panel shows the whole range of dose applied (0.01 Gy to 8.55 Gy); the right panel shows the low dose range up to 0.1 Gy. Figure S3: Relative DPP dependence of the microSilicon (60023), the Diode E (60017) and the microDiamond (60019) presented as the ratio of the detector’s signal and the Semiflex ionization chamber’s signal that has been corrected for recombination loss. Figure S4: Comparison between the cross‐plane profiles measured using microSilicon, Diode E and microDiamond at a Varian TrueBeam linear accelerator using a field size of 0.6 × 0.6 cm² (left) and 1.6 × 1.6 cm² (right). The percentage deviations of the microSilicon and Diode E from the microDiamond’s profile are shown at the bottom. Table S1: Fitting parameters for K(x) according to eq. (S2) for the microSilicon (60023) and the Diode E (60017). σi in mm. [file MP-46-4257-s001.pdf]

Supplementary Materials to the Publication:

Technical Note: Characterization of the new microSilicon diode detector

Ann-Britt Schönfeld[1], Daniela Poppinga[2], Rafael Kranzer[2], Rudy Leon De Wilde[3], Kay Willborn[4], Björn Poppe[1], Hui Khee Looe[1]

[1] University Clinic for Medical Radiation Physics, Medical Campus Pius Hospital, Carl von Ossietzky University, Oldenburg, Germany

[2] PTW-Freiburg, Freiburg, Germany

[3] University Clinic for Gynecology, Pius Hospital, Oldenburg, Germany

[4] Clinic for Radiation Therapy, Pius Hospital, Oldenburg, Germany

Corresponding author: ann-britt.ulrichs@uol.de

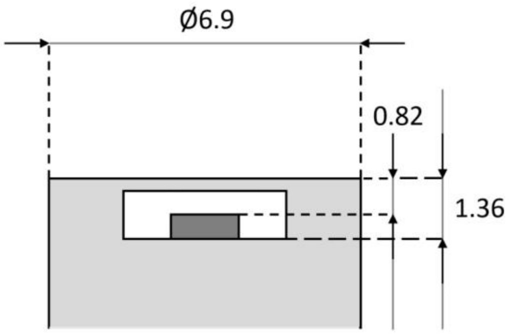

Figure S1: Schematic cross-section of the microSilicon. All dimensions given in the unit mm. The silicon chip is shown in dark grey, the detector housing in light grey and the epoxy encapsulation in white.

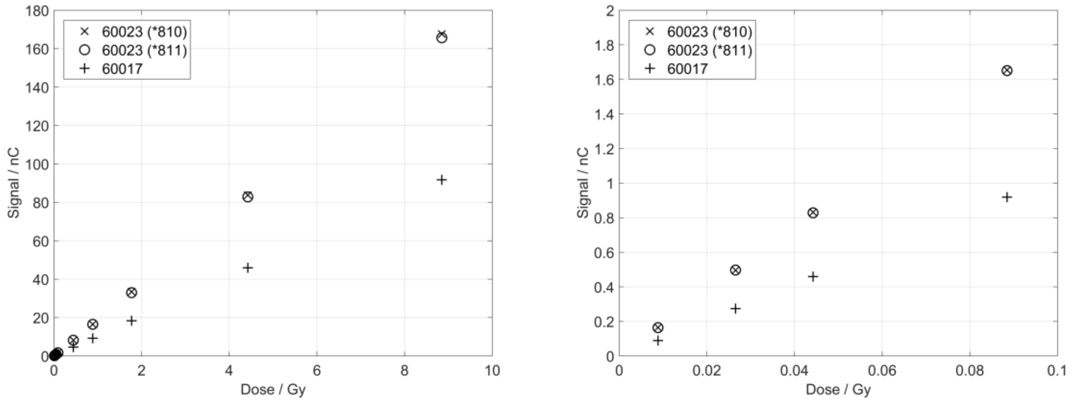

Figure S2: Linearity of the dose response of the microSilicon (60023) and Diode E (60017). The left panel shows the whole range of dose applied (0.01 Gy to 8.55 Gy); the right panel shows the low dose range up to 0.1 Gy.

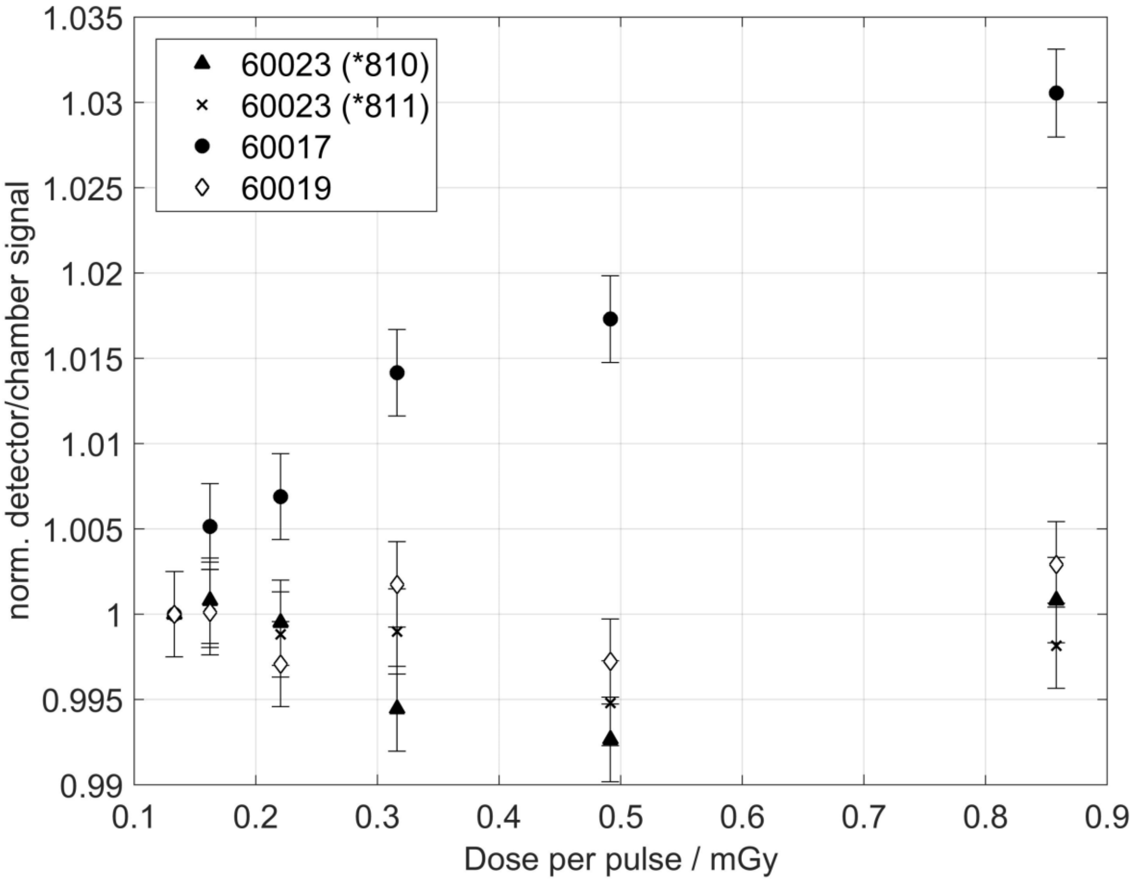

Figure S3: Relative DPP dependence of the microSilicon (60023), the Diode E (60017) and the microDiamond (60019) presented as the ratio of the detector's signal and the Semiflex ionization chamber's signal that has been corrected for recombination loss.

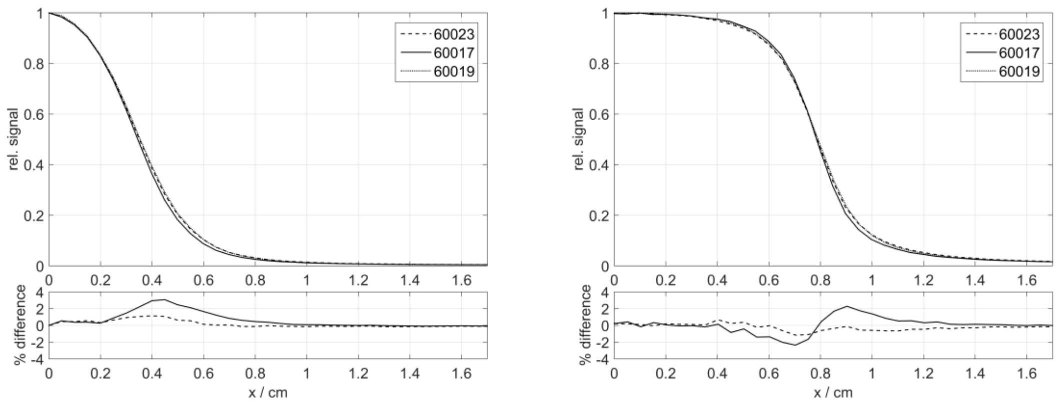

Figure S4: Comparison between the cross-plane profiles measured using microSilicon, Diode E and microDiamond at a Varian TrueBeam linear accelerator using a field size of 0.6 x 0.6 cm² (left) and 1.6 x 1.6 cm² (right). The percentage deviations of the microSilicon and Diode E from the microDiamond's profile are shown at the bottom.

Derivation of the *K(x)* according to eq. (1)

Both the signal profiles *M(x)* and the dose profile *D(x)* in eq. (1) were fitted by sums of three centered one-dimensional Gaussian functions and then Fourier transformed. The deconvolution was performed by taking the quotient of these Fourier transforms

$$\mathrm{FT}[K(x)] = \frac{1}{\sqrt{2\pi}} \frac{\mathrm{FT}[M(x)]}{\mathrm{FT}[D(x)]} \quad \text{(S1)}$$

The result in eq. (S1) was fitted by a sum of five centered Gaussian functions and then the inverse Fourier transformation was performed analytically. The resulting *K(x)* is approximated by a sum of five centered one-dimensional Gaussian functions according to eq. (S2)

$$K(x) = \sum_{i=1}^{5} \frac{A_{i,K}}{\sigma_{i,K}} exp\left(-\frac{x^2}{2\sigma^2{}_{i,K}}\right) \quad \text{(S2)}$$

with the normalization factors $A_{i,K}$ and the standard deviations $\sigma_{i,K}$ being the detector specific free parameters.

Table S1: Fitting parameters for *K(x)* according to eq. (S2) for the microSilicon (60023) and the Diode E (60017). σ_i in mm.

	$A_{1,K}$	$A_{2,K}$	$A_{3,K}$	$A_{4,K}$	$A_{5,K}$	$\sigma_{1,K}$	$\sigma_{2,K}$	$\sigma_{3,K}$	$\sigma_{4,K}$	$\sigma_{5,K}$
60023	0.012	-33.520	33.360	0.532	0.001	5.401	1.199	1.201	0.464	0.141
60017	-3.970	-0.495	4.962	-0.105	-0.006	0.294	0.269	0.296	1.190	-1.189
